# A multicenter matched case-control analysis on seven polymorphisms from *HMGB1* and *RAGE* genes in predicting hepatocellular carcinoma risk

**DOI:** 10.18632/oncotarget.15202

**Published:** 2017-02-08

**Authors:** Dan Wang, Xiaoying Qi, Fang Liu, Chuanhua Yang, Wenguo Jiang, Xiaodan Wei, Xuri Li, Jia Mi, Geng Tian

**Affiliations:** ^1^ Medicine and Pharmacy Research Center, Binzhou Medical University, Yantai, Shandong, China

**Keywords:** hepatocellular carcinoma, HMGB1/RAGE axis, polymorphism, genetic susceptibility, haplotype

## Abstract

Based on 540 hepatocellular carcinoma patients and 540 age- and gender-matched controls, we tested the hypothesis that high mobility group protein box1 (*HMGB1*) and the receptor for advanced glycation end products (*RAGE*) genes are two potential candidate susceptibility genes for hepatocellular carcinoma in a multicenter hospital-based case-control analysis. The genotypes of seven widely-studied polymorphisms were determined, and their distributions respected the Hardy-Weinberg equilibrium. The mutant alleles of two polymorphisms, rs1045411 in *HMGB1* gene and rs2070600 in *RAGE* gene, had significantly higher frequencies in patients than in controls (*P* < 0.001), with the power to detect this significance of being over 99.9%. Moreover, the above two polymorphisms increased the risk of developing hepatocellular carcinoma significantly, particularly for rs2070600 under the additive (odds ratio [OR] = 1.77; 95% confidence interval [CI]: 1.34-2.32; *P* < 0.001) and dominant (OR = 1.75; 95% CI: 1.23-2.50; *P* = 0.002) models after adjusting for body mass index, smoking and drinking. Haplotype analysis showed that the T-C-T haplotype (rs1045411-rs2249825-rs1415125) in *HMGB1* gene was associated with a 2.47-fold (95% CI: 1.41-4.34; *P* = 0.002) increased risk of hepatocellular carcinoma compared with the commonest C-C-T haplotype after adjustment. In *RAGE* gene, the T-T-A-G (rs1800625-rs1800624-rs2070600-rs184003) (adjusted OR; 95% CI; P: 1.75; 1.02-3.03; 0.045) and T-T-A-T (adjusted OR; 95% CI; P: 1.95; 1.01-3.76; 0.048) haplotypes were associated with a marginally increased risk of hepatocellular carcinoma compared with the commonest T-T-G-G haplotype. In summary, we identified two risk-associated polymorphisms (rs1045411 and rs2070600), and more importantly a joint impact of seven polymorphisms from the HMGB1/RAGE axis in susceptibility to hepatocellular carcinoma.

## INTRODUCTION

The importance of the ligand-receptor axis involving high mobility group protein box1 (HMGB1) and the receptor for advanced glycation end products (RAGE) in inflammation and tumorigenesis has been increasingly recognized by many experimental and clinical investigators [1–4]. HMGB1 is a nuclear DNA-binding factor that exerts an important regulatory role in chromatin architecture and transcriptional regulation [5]. The binding of HMGB1 to RAGE can stimulate the expression of Ras, PI3K, and Rho, which are major downstream signaling molecules responsible for the NF-κB activation [6, 7]. Studies in growing numbers have reported the concordant over-expression of HMGB1 and RAGE in a variety of cancer types, including hepatocellular carcinoma [8–11]. Several lines of clinical evidence suggested that HMGB1 and RAGE possessed the potentials to serve as powerful prognostic and therapeutic targets for hepatocellular carcinoma [12–14]. The genes coding for *HMGB1* and *RAGE* mapped on different chromosomes carry many polymorphic loci. There is compelling evidence that circulating variation of HMGB1 and RAGE is predominantly under genetic control [15–17]. One therefore could hypothesize that *HMGB1* and *RAGE* genes are two potential candidate susceptibility genes for hepatocellular carcinoma.

To test this hypothesis, we designed a multicenter case-control study and genotyped seven widely-studied polymorphisms from *HMGB1* and *RAGE* genes among 540 hepatocellular carcinoma patients and 540 age- and gender-matched controls.

## RESULTS

### Basic characteristics

The summarization and comparison of basic characteristics between 540 patients and 540 matched controls are shown in Table 1. Patients were matched with controls on age and gender. The mean level of BMI was comparable between patients and controls (*P* = 0.372). By contrast, the percentages of current or ever cigarette smokers and alcohol drinkers were significantly higher in patients than in controls (*P* < 0.001).

**Table 1:** The summarization and comparison of basic characteristics between 540 patients and 540 age- and gender-matched controls

Characteristics	Patients	Controls	P*
Age (years)	51.5 (6.7)	50.4 (6.8)	0.165
Male gender	61.7%	61.3%	0.900
BMI (kg/m^2^)	25.4 ± 2.2	25.7 ± 2.0	0.372
Current/ever smokers	58.9%	33.9%	<0.001
Current/ever alcohol drinkers	63.9%	34.4%	<0.001

BMI: body mass index. Quantitative data are listed as the mean ± standard deviation, and categorical data are listed as the percentage. Quantitative and categorical data are compared between patients and controls with the t test and Chi-squared test, respectively.

### Single polymorphism analysis

**Figure 1:**
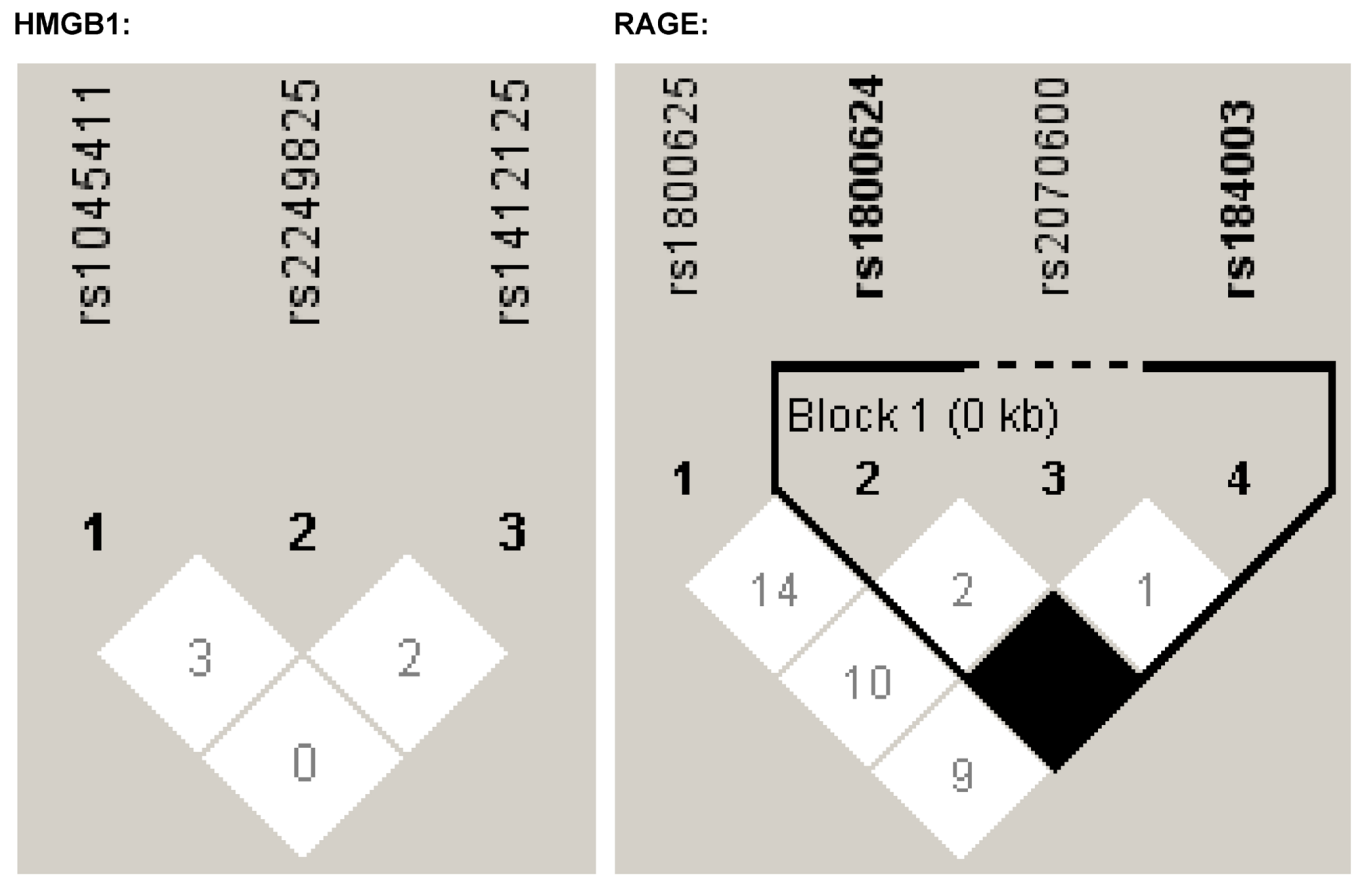
The linkage patterns of polymorphisms in *HMGB1* and *RAGE* genes

The genotypes and alleles of seven polymorphisms under study are compared in Table 2. There was no significant deviation from the Hardy-Weinberg equilibrium for all seven polymorphisms in control subjects (all *P* > 0.05), and their linkage patterns within each gene are illustrated in Figure 1. The mutant alleles of two polymorphisms, rs1045411 in *HMGB1* gene and rs2070600 in *RAGE* gene, had significantly higher frequencies in hepatocellular carcinoma patients than in controls (both *P* < 0.001), and this significance was attained even after the Bonferroni correction (*P* < 0.05/7). Further, the power to detect the significance was 99.6% and 99.9% respectively for allele comparisons of rs1045411 and rs2070600 between patients and controls. In addition, the genotypes of rs1800625 differed significantly between the two groups (*P* = 0.008), while its allele distributions were only marginally significant (*P* = 0.034) that did not survive the Bonferroni correction. The other polymorphisms showed no statistical significance in susceptibility to hepatocellular carcinoma.

**Table 2:** The comparison of the genotypes and alleles of seven polymorphisms from *HMGB1* and *RAGE* genes between 540 patients and 540 age- and gender-matched controls

Polymorphisms	w/m	Group	Genotype			Allele	P_genotype_	P_allele_
			ww	wm	mm	m		
rs1045411	C/T	Patients	349	158	33	20.82%	<0.001	<0.001
		Controls	405	127	8	13.26%		
rs2249825	C/G	Patients	349	168	23	19.84%	0.521	0.513
		Controls	354	170	16	18.75%		
rs1415125	T/C	Patients	273	216	51	29.38%	0.588	0.272
		Controls	290	205	45	27.27%		
rs1800625	T/C	Patients	403	107	30	15.37%	0.008	0.034
		Controls	417	113	10	12.31%		
rs1800624	T/A	Patients	374	147	19	17.12%	0.610	0.999
		Controls	370	155	15	17.05%		
rs2070600	G/A	Patients	254	202	84	34.24%	<0.001	<0.001
		Controls	321	190	29	22.92%		
rs184003	G/T	Patients	277	221	42	28.21%	0.194	0.064
		Controls	303	207	30	24.81%		

ww: homozygous wild genotype; wm: heterozygous genotype; mm: homozygous mutant genotype; m: mutant allele. Genotypes and alleles were compared between patients and controls with the Chi-squared test.

The risk prediction of each polymorphism under study for hepatocellular carcinoma is shown in Table 3. After adjusting for body mass index (BMI), smoking and drinking, only rs1045411 and rs2070600 polymorphisms were significantly associated with an increased risk of hepatocellular carcinoma, particularly for rs2070600 under both genetic models. In detail, the mutant allele carriers of rs2070600 had a 1.77-fold (95% CI: 1.34-2.32; *P* < 0.001) and 1.75-fold (95% CI: 1.23-2.50; *P* = 0.002) increased risk after adjustment under the additive and dominant models, respectively. There was no statistical significance for the other five polymorphisms under study.

**Table 3:** The risk prediction of seven polymorphisms from *HMGB1* and *RAGE* genes for hepatocellular carcinoma before and after adjusting for confounders under the additive and dominant models

Polymorphisms	Additive model		Dominant model	
	Unadjusted	Adjusted	Unadjusted	Adjusted
rs1045411	1.67; 1.21-2.31; 0.002	1.64; 1.18-2.29; 0.003	1.64; 1.13-2.40; 0.010	1.62; 1.10-2.39; 0.014
rs2249825	1.07; 0.79-1.46; 0.654	1.00; 0.73-1.37; 0.997	1.04; 0.73-1.49; 0.822	0.95; 0.66-1.38; 0.784
rs1415125	1.10; 0.85-1.44; 0.460	1.12; 0.85-1.47; 0.414	1.14; 0.81-1.60; 0.464	1.13; 0.80-1.61; 0.482
rs1800625	1.25; 0.90-1.74; 0.183	1.20; 0.85-1.68; 0.295	1.15; 0.77-1.72; 0.493	1.08; 0.71-1.63; 0.718
rs1800624	1.01; 0.73-1.39; 0.974	1.02; 0.73-1.41; 0.919	0.97; 0.67-1.40; 0.863	0.96; 0.66-1.40; 0.831
rs2070600	1.67; 1.28-2.17; <0.001	1.77; 1.34-2.32; <0.001	1.65; 1.17-2.33; 0.005	1.75; 1.23-2.50; 0.002
rs184003	1.19; 0.90-1.58; 0.211	1.20; 0.90-1.59; 0.215	1.21; 0.86-1.71; 0.282	1.21; 0.85-1.72; 0.295

OR: odds ratio; 95% CI: 95% confidence interval. *P was calculated after adjusting for body mass index, smoking and drinking under the conditional binary Logistic regression model. Additive model was defined as homozygous wild genotype *vs*. heterozygous genotype vs. homozygous mutant genotype. Dominant model was defined as homozygous wild genotype *vs*. heterozygous genotype plus homozygous mutant genotype.

### Haplotype analysis

Haplotype analysis was separately conducted in *HMGB1* and *RAGE* genes in view of different chromosomes they mapped on, and the corresponding results are shown in Table 4. As the low-penetrance haplotypes usually carry a high risk of producing chance false-positive findings, haplotype analysis was only restricted to the common haplotypes, which had an estimated frequency of at least 3% in both patients and controls.

**Table 4:** The comparison of derived common haplotypes based on seven polymorphisms from *HMGB1* and *RAGE* genes between 540 patients and 540 age- and gender-matched controls, and their risk prediction for hepatocellular carcinoma

Haplotypes	Patients	Controls	*P*	Crude OR; 95% CI; P	Adjusted OR; 95% CI; P*
**HMGB1 gene (rs1045411-rs2249825-rs1415125)**					
C-C-T	45.01%	53.84%	0.013	Reference haplotype	
C-C-C	17.18%	17.79%	0.632	1.14; 0.77-1.69; 0.516	1.18; 0.79-1.76; 0.428
T-C-T	12.59%	5.56%	0.003	2.55; 1.44-4.5; 0.001	2.47; 1.41-4.34; 0.002
C-G-T	10.66%	9.71%	0.946	1.26; 0.76-2.11; 0.371	1.18; 0.7-1.98; 0.544
C-G-C	6.33%	5.42%	0.466	1.39; 0.72-2.66; 0.321	1.27; 0.66-2.47; 0.476
T-C-C	5.38%	4.07%	0.087	1.48; 0.75-2.95; 0.262	1.46; 0.73-2.92; 0.278
**RAGE gene (rs1800625-rs1800624-rs2070600-rs184003)**					
T-T-G-G	30.43%	39.43%	0.002	Reference haplotype	
T-T-G-T	16.02%	18.29%	0.482	1.16; 0.75-1.78; 0.502	1.22; 0.78-1.9; 0.382
T-T-A-G	13.98%	11.99%	0.118	1.53; 0.9-2.58; 0.116	1.75; 1.02-3.03; 0.045
T-A-G-G	10.64%	10.11%	0.615	1.36; 0.81-2.29; 0.243	1.44; 0.84-2.46; 0.182
T-T-A-T	7.44%	4.82%	0.032	1.88; 0.99-3.6; 0.056	1.95; 1.01-3.76; 0.048
C-T-G-G	5.93%	4.82%	0.785	1.43; 0.73-2.8; 0.293	1.41; 0.71-2.79; 0.326

OR: odds ratio; 95% CI: 95% confidence interval. *P was calculated after adjusting for body mass index, smoking and drinking.

In *HMGB1* gene, the frequency of T-C-T haplotype (rs1045411-rs2249825-rs1415125, similarly hereinafter) was significantly higher in patients than in controls (12.59% vs. 5.56%, *P* = 0.003) with the study power reaching 99.9%, and this haplotype was associated with a 2.47-fold (95% CI: 1.41-4.34; *P* = 0.002) increased risk of hepatocellular carcinoma after adjusting for BMI, smoking and drinking when compared with the commonest haplotype C-C-T.

In *RAGE* gene, the commonest T-T-G-G haplotype (rs1800625-rs1800624-rs2070600-rs184003, similarly hereinafter) was frequently seen in controls compared with patients, and relative to this haplotype, T-T-A-G (OR; 95% CI; P: 1.75; 1.02-3.03; 0.045) and T-T-A-T (OR; 95% CI; P: 1.95; 1.01-3.76; 0.048) haplotypes were associated with a marginally increased risk of hepatocellular carcinoma after adjusting for confounders mentioned above, with the study power of 28.0% and 71.9%, respectively.

## DISCUSSION

In this multicenter matched case-control study, we set out to examine the hypothesis that *HMGB1* and *RAGE* genes are two potential candidate susceptibility genes for hepatocellular carcinoma. Through extensive analyses, our findings support this hypothesis by identifying two risk associated polymorphisms, rs1045411 and rs2070600 from the HMGB1/RAGE axis, and more importantly a joint impact of seven polymorphisms in susceptibility to hepatocellular carcinoma.

Hepatocellular carcinoma is one of the common solid malignancies in the world, and it is particularly prevalent in China [18, 19]. Many epidemiological studies have suggested that a strong genetic component underlies the hepatocarcinogenesis [20, 21]. Although genome-wide association studies are widely utilized to characterize the genetic profiles of hepatocellular carcinoma, the results seem far from promising [22, 23]. Given the ubiquity of genetic heterogeneity, it is of special importance to develop a catalogue of cancer-susceptibility genes or polymorphisms within individual ethnic groups. Bearing this in mind, we selected seven well-characterized polymorphisms from *HMGB1* and *RAGE* genes to assess their genetic susceptibility to hepatocellular carcinoma in a large Han Chinese population.

First, it is interesting to notice a leading role played by rs1045411 and rs2070600 polymorphisms that conferred a significant increased risk for hepatocellular carcinoma, and the two polymorphisms were respectively resided in *HMGB1* and *RAGE* genes. On one hand, the polymorphism rs1045411, which is located in the 3'-untranslated region of *HMGB1*, might be biologically functional as the mutant allele of this polymorphism was recently reported to be significantly associated with higher positive blood culture rates and elevated cytokine levels, particularly in patients with chronic lung diseases or diabetes as co-morbidities [24]. So far the physiological significance of rs1045411 is uncertain, and the location of this polymorphism could suggest a role in mRNA stability as microRNAs can bind the 3'-untranslated regions of mRNA transcripts and inhibit gene expression at the posttranscriptional level. Moreover, in the present study, the mutant allele of rs1045411 was associated with a significantly increased risk of hepatocellualr carcinoma, while another study in a Taiwan population found that carriers of this mutant allele had a lower risk [25], likely due to differences in lifestyles, diets or study power. On the other hand, the polymorphism rs2070600 is non-synonymous (Gly82Ser) in the exon 3 of *RAGE* gene, and the association of this polymorphism was significant with a wide range of cancer types, including lung cancer [26], breast cancer [27], ovarian cancer [28] and colorectal cancer [29]. However, a recent study by Su et al failed to support this significant association in Taiwanese, and instead they found that another promoter polymorphism rs1800625 in *RAGE* gene was linked to the increased risk for hepatocellular carcinoma [30]. Besides genetic profiles, the pathogenesis of hepatocellular carcinoma is also closely related to environmental, dietary, life style factors, which are sharply different between northern Han Chinese in the present study and Taiwanese. It remains possible to speculate that if involved, the rs2070600 mutation might account for circulating variation of soluble RAGE, which was demonstrated to play a protective role in the development of cancer by a meta-analysis [16].

Second, it is widely recognized that single locus may not by itself have a significant association with a disease because its effect may be small and dependent on the nearby loci that compensate for variation in the locus under study [31]. To address this claim, we performed the haplotype analysis, and it is worth noting for a joint impact of multiple polymorphisms from the HMGB1/RAGE axis on the hepatocarcinogenesis. This finding is understandable in view of the fact that the role of any given locus in the course of hepatocellular carcinoma is likely to be small on average. Nevertheless, this study provides preliminary evidence that potentially unfavorable combinations of polymorphisms from the HMGB1/RAGE axis could synergistically affect the onset and progression of hepatocellular carcinoma.

Finally, there are several limitations to this study. Firstly, this is a multicenter case-control study, and the probability of population stratification and admixture might be high. However, the conformity of seven polymorphisms to the Hardy-Weinberg equilibrium can somewhat reduce this probability. Secondly, the total sample size of the present study (*N* = 1080) is not large enough to justify the impact of low-penetrance alleles or genotypes. However, in case of significant findings in the present study, the statistical power to detect a significant allele difference between patients and controls was over 99%, indicating the somewhat validity of our association analyses. Thirdly, because this study was carried out in a Han Chinese population, we are reticent in extrapolating our chief conclusions to the other races or ethnicities, as it is widely recognized that strong genetic heterogeneity underlies the development of hepatocellular carcinoma [32]. Fourthly, although patients and controls were matched on age and gender at enrolment, other lifestyle factors (such as smoking and drinking habits) different remarkably between the two groups, and clinical markers are not available for us to analyze, leaving some room for further explorations especially from the aspect of gene-to-environment interaction. We agree that a large-scale well-designed study is warranted to overcome these limitations and produce convincing evidence.

In summary, we identified two risk-associated polymorphisms (rs1045411 and rs2070600) and more importantly a joint impact of seven polymorphisms from the HMGB1/RAGE axis in susceptibility to hepatocellular carcinoma. From a broader clinical perspective, a greater understanding of this axis is critical to furthering our understanding of the genetic underpinnings of hepatocellular carcinoma or related solid tumors.

## MATERIALS AND METHODS

### Study subjects

All study subjects were gathered from three local hospitals (Yuhuangding Hospital, Yantai Affiliated Hospital of Binzhou Medical University and Shandong Provincial Hospital) in Shandong province, China between February 2014 and September 2016, and they gave informed, written consent prior to participation. The protocols of this study were approved by the ethics committees at all participating hospitals.

### Basic characteristics

The clinical diagnosis of hepatocellular carcinoma was confirmed by histopathological examinations. Hepatocellular carcinoma patients (*n* = 540) were matched on age (within 2-year strata) and gender with 540 cancer-free controls gathered from the same hospital that had no self-reported family history of any malignancies excluding non-melanoma skin cancer within three generations. For patients, age was recorded for the initial onset age of hepatocellular carcinoma. Besides baseline age and gender, we also recorded body height, body weight, tobacco smoking and alcohol drinking. Body height and weight were measured in light clothing, and body mass index (BMI) was calculated as weight in kilometer divided by height in meters-squared. Tobacco smokers refer to the current or ever smokers (at least ten cigarettes per week) who smoked more than 3 months. Alcohol drinkers refer to the current or ever drinkers (at least one time a week) who drank more than three months.

At the time of enrolment, each study subject provided a fasted peripheral venous blood sample, which was centrifuged within the same day at 3000×g for 5 min at room temperature, and then was kept at minus 40°C temperature until batch assayed.

### Polymorphism selection

From *HMGB1* gene, three polymorphisms including rs1045411 (3’-untranslated region), rs2249825 (promoter region) and rs1415125 (promoter region) were selected. From *RAGE* gene, four polymorphisms including rs1800625 (promoter region), rs1800624 (promoter region), rs2070600 (exon 3) and rs184003 (intron 7) were selected. These polymorphisms were widely studied in predisposition to a variety of cancer types, including breast cancer [33], gastric cancer [34, 35], colorectal cancer [29, 36], lung cancer [37, 38] and so on.

### Genotype determination

Genomic DNA was prepared from white blood cells, and was extracted by either a phenol-chloroform method or a salting-out method. Genotypes of seven polymorphisms under study were determined by the popular ligase detection reaction method, as previously reported by Niederhauser and coworker [39]. The accuracy of this genotyping method was validated by re-genotyping 10% (*N* = 108) of all DNA samples through a random selection manner. The genotyping results of these 108 duplicated samples fitted perfectly.

### Statistical analysis

Quantitative data were summarized as the mean ± standard deviation, and categorical data were summarized as the percentage. The Hardy-Weinberg equilibrium refers to the consistency of genotype frequencies with the two alleles being independently sampled from a population of alleles, and it was tested by the Chi-squared test in controls to avoid gross genotyping misclassifications. Genetic inheritance pattern of each polymorphism was assessed to be either additive (wild homozygote vs. heterozygote vs. mutant homozygote) or dominant (wild homozygote vs. combined heterozygote and mutant homozygote). A multivariate conditional binary Logistic regression analysis was conducted to model individual polymorphisms after considering the confounding impact of BMI, smoking and drinking. Further haplotype analysis was performed to examine possible joint impact of individual polymorphisms within the same gene after adjustment. In theory, a haplotype is defined as a combination of two or more alleles on a single chromosome. The frequencies of derived haplotypes were compared between patients and controls, and their risk prediction for hepatocellular carcinoma was calculated after adjusting for BMI, smoking and drinking. All tests are two-sided, and *P* < 0.05 was considered statistically significant unless otherwise indicated. Above calculations were performed with the R software (v3.3.1), which can be freely downloaded from the website https://www.r-project.org/. Haplotype analyses were performed with the HAPLO.STATS program (v1.7.1) realized under the R environment (v3.3.1). The HAPLOTYPE.STATS program provides different methods to testing the association of haplotypes with traits after adjustment for effects from other confounders, which has a statistical advantage to further the understanding of the roles of different haplotypes [40, 41]. Linkage disequilibrium patterns between polymorphisms within the same gene was assessed by calculating D'/LOD (LOD is the log of the likelihood odds ratio, a measure of confidence in the value of D') with the Haploview (v4.2). Study power was estimated with the CaTS software (http://www.sph.umich.edu/csg/abecasis/CaTS/index.html).
